# Descriptive epidemiology of rubella disease and associated virus strains in Uganda

**DOI:** 10.1002/jmv.25604

**Published:** 2019-10-09

**Authors:** Phionah Tushabe, Josephine Bwogi, Emily Abernathy, Molly Birungi, James P. Eliku, Ronald Seguya, Henry Bukenya, Prossy Namuwulya, Proscovia Kakooza, Suganthi Suppiah, Theopista Kabaliisa, Mayi Tibanagwa, Immaculate Ampaire, Annet Kisakye, Andrew Bakainaga, Charles R. Byabamazima, Joseph P. Icenogle, Barnabas Bakamutumaho

**Affiliations:** ^1^ EPI Laboratory Uganda Virus Research Institute Entebbe Uganda; ^2^ Division of Viral Diseases, National Center for Immunization and Respiratory Diseases United States Centers for Disease Control and Prevention Atlanta Georgia; ^3^ Ministry of Health Uganda Kampala Uganda; ^4^ World Health Organization Kololo Uganda; ^5^ WHO Inter‐Country Support Team Office For Eastern and Southern Africa (IST/ESA) Harare Zimbabwe

**Keywords:** congenital rubella syndrome, genotype, vaccination

## Abstract

Rubella virus causes a mild disease; however, infection during the first trimester of pregnancy may lead to congenital rubella syndrome (CRS) in over 80% of affected pregnancies. Vaccination is recommended and has been shown to effectively reduce CRS incidence. Uganda plans to introduce routine rubella vaccination in 2019. The World Health Organization recommends assessing the disease burden and obtaining the baseline molecular virological data before vaccine introduction. Sera collected during case‐based measles surveillance from January 2005 to July 2018 were tested for rubella immunoglobulin M (IgM) antibodies. Sera from confirmed rubella outbreaks from January 2012 to August 2017 were screened using real‐time reverse‐transcription polymerase chain reaction (RT‐PCR); for positive samples, a region within the E1 glycoprotein coding region was amplified and sequenced. Of the 23 196 suspected measles cases serologically tested in parallel for measles and rubella, 5334 (23%) were rubella IgM‐positive of which 2710 (50.8%) cases were females with 2609 (96.3%) below 15 years of age. Rubella IgM‐positive cases were distributed throughout the country and the highest number was detected in April, August, and November. Eighteen (18%) of the 100 sera screened were real‐time RT‐PCR‐positive of which eight (44.4%) were successfully sequenced and genotypes 1G and 2B were identified. This study reports on the seroprevalence and molecular epidemiology of rubella. Increased knowledge of former and current rubella viruses circulating in Uganda will enhance efforts to monitor the impact of vaccination as Uganda moves toward control and elimination of rubella and CRS.

## 1 INTRODUCTION

Rubella is caused by an RNA virus belonging to genus *Rubivirus* and family *Matonaviridae* (formerly belonged to *Togaviridae*),^1^ and usually presents with mild disease with up to 50% of cases being asymptomatic.^2^, ^3^ Rubella is characterized by a maculopapular rash and fever. The symptoms can mimic other febrile rash illnesses, including measles, necessitating laboratory confirmation. Although generally mild, infection in susceptible women during early pregnancy commonly results in significant, preventable disease burden in the form of abortion, stillbirth, spontaneous abortion, premature birth, and adverse outcomes in the infant including mental retardation, visual and hearing impairments, and/or congenital heart defects collectively termed congenital rubella syndrome (CRS). The global estimates of children born with CRS in 2008 exceeded 110 000 with these estimates suggesting that the highest burden was in South East Asian (approximately 48% of total CRS cases) and African (approximately 38%) regions.^4^, ^5^ Disease burden attributable to rubella virus is preventable through vaccination.^6^ While rubella‐containing vaccines have been available since 1969^3^ either as monovalent or as combined formulations, it is only now that Uganda plans to introduce a rubella‐containing vaccine into the routine schedule. Prevaccine estimates on the rubella disease burden, epidemiology, and associated virus strains are a standard requirement and recommendation by the World Health Organization (WHO)^7^ primarily to guide country's decision making for the vaccine introduction, ease of monitoring vaccine effectiveness, and better understanding of virus transmission.

National case‐based surveillance of measles, and indirectly rubella, in Uganda started in 2003 with samples from all suspected measles cases being tested for antimeasles and antirubella immunoglobulin M (IgM) antibodies in parallel. Despite this, documented knowledge of the burden of rubella in the country is limited to only two studies.^8^, ^9^ With such limited published data, the trends of rubella incidence in the country are not available for program use and the impact after vaccine introduction may be poorly estimated because of the lack of baseline data.

Collection of specific specimens for molecular virological data is challenging as it requires timely collection of throat swab and oral fluid specimens by experienced personnel and a stringent reverse cold chain. These requirements are often hard to achieve through the routine surveillance for measles and rubella. Alternative samples such as serum have been shown to provide molecular virological data retrospectively^10^, ^11^ and are a potential source of baseline data on the genetic diversity of the rubella viruses in the prevaccine era. Rubella virus RNA may be detected in acute phase serum specimens (0‐3 days from rash onset) that are rubella IgM‐positive by using the polymerase chain reaction and positive samples can be genotyped.^10^, ^11^

We report on the rubella disease burden from January 2005 to July 2018 using case‐based measles surveillance data as well as the molecular virological data using archived serum samples from confirmed rubella outbreaks from January 2012 to August 2017. This documentation will provide baseline information on the epidemiology of rubella before rubella vaccine introduction in Uganda.

## 2 MATERIALS AND METHODS

The Regional Measles Reference laboratory in Uganda has been a member of the Global WHO Measles and Rubella Laboratory Network^12^ since 2000 and the rubella cases in this study were identified through measles case‐based surveillance following guidelines provided by the WHO Regional Office for Africa.^13^ Based on these guidelines, a suspected measles case is defined as “any person with generalized maculopapular rash and fever plus one of the following, cough or coryza (runny nose) or conjunctivitis (red eyes) or any person in whom a clinician suspects measles.” Individuals fitting these criteria and who presented at health facilities within 30 days of rash onset were investigated using a standard case investigation form for demographic and clinical history data and a serum specimen collected for laboratory analysis. When five or more cases were investigated in a health facility or district in a month, this was classified as a suspected outbreak. Specimens were transported to the laboratory under cold chain.

### 2.1 Enzyme‐linked immunosorbent assay

Once in the laboratory, sera were tested by both the Enzygnost® Anti‐Measles Virus/IgM and Enzygnost® Anti‐Rubella Virus/IgM ELISAs (Dade Behring, Germany from 2000 to 2006 renamed Siemens, Marburg, Germany from 2006 to 2017) in parallel within 7 days of receipt. As there was no rubella vaccine, all rubella IgM‐positive cases were classified as laboratory‐confirmed rubella. A rubella outbreak was confirmed when three of the five cases investigated as a suspected outbreak tested rubella IgM‐positive.

### 2.2 Molecular testing

#### 2.2.1 Sample selection, RNA extraction, and real‐time reverse‐transcription polymerase chain reaction (RT‐PCR)

Serum samples from confirmed rubella outbreaks collected between January 2012 and August 2017 that tested rubella IgM‐positive and had been collected 0 to 2 days from rash onset were selected for this study. These samples that were being stored at −20°C were retrieved from storage, thawed, and 400 µl aliquots shipped to the Centers for Disease Control (CDC) and Prevention, Atlanta, GA, on dry ice. At CDC, RNA was extracted using 140 µl of the serum and the QIAamp viral RNA extraction kit (Qiagen, Germantown, MD), following the manufacturer's instructions and were screened for rubella virus RNA using a SuperScript III real‐time RT‐PCR kit (Invitrogen, Carlsbad, CA) and primer/probe mix (RV11, RV12, RV12‐2 primers and probe and RNAse P forward and reverse primers and probe) developed by CDC.^9^, ^14^ Specimens with *C*_t_ values below 40 were considered positive and used for genotyping.

#### 2.2.2 Genotyping and sequencing assays

RNA from all real‐time RT‐PCR positives were added to a nested RT‐PCR assay with Superscript III One‐Step Platinum Taq kit (Invitrogen) as described by Pukuta et al.^11^ In brief, 2.5 µl of the extracted RNA was run in a 25 µl reaction containing 12.5 µl of 2× reaction buffer, 0.25 µl of each of the forward and reverse primers (Table 1), 5 µl of betaine (Sigma‐Aldrich, St. Louis, MO), 0.25 µl of RNase inhibitor, and 0.5 µl of Superscript III Platinum Taq enzyme mix at final concentrations of 1×, 0.2 µM, 1M, 0.4 U, and 0.1 U respectively. Cycling conditions for the 1st round RT‐PCR consisted of 30 minutes at 55°C, 2 minutes at 94°C and 40 cycles of 10 seconds at 94°C, 15 seconds at 55°C and 1 minute at 68°C. For the 2nd round, 1 µl of the 1st round PCR product was used with the primers in Table 1 and the initial transcription step eliminated from the cycling conditions.

**Table 1:** 1st and 2nd round RT‐PCR primer sequences

Name	PCR	Size	Sequence	Nucleotides
8633F	1st round	20 nt	5′‐AGC GAC GCG GCC TGC TGG GG‐3′	8633‐8652
9577R		21 nt	5′‐CGC CCA GGT CTG CCG GGT CTC‐3′	9557‐9577
8669F	2nd round	20 nt	5′‐GTG ATG AGC GTG TTC GCC CT‐3′	8669‐8688
9541R		21 nt	5′‐GTG TGT GCC ATA CAC CAC GCC‐3′	9521‐9541

Abbreviation: RT‐PCR, reverse‐transcription polymerase chain reaction.

The resulting products were purified using the ExoSAP‐IT PCR Product Cleanup Reagent (Thermo Fisher Scientific, Carlsbad, CA). Briefly, 5 µl of the PCR product was mixed with 2 µl of the enzyme and incubated at 37°C for 4 minutes followed by a 1‐minute incubation at 80°C in a thermal cycler. The 739‐nt sequences required for the genotyping of rubella viruses were determined bidirectionally with an Applied Biosystems Prism BigDye Terminator Cycle Sequencing Ready Reaction Kit (Applied Biosystems, Austin, TX) and a 3500 DNA sequencer (Applied Biosystems, Japan). To cover the sequence of the 739‐nt region, the primers from the second round PCR and two internal primers (8945F and 9112R^9^) were used.

#### 2.2.3 Phylogenetic analyses

Analyses of the sequences were performed using Sequencher version 4.10.1 (Gene Codes Corporation, Ann Arbor, MI) and the genotypes determined by comparison with the 32 WHO rubella virus reference sequences.^15^ Sublineage reference sequences (rubella virus genetic grouping nomenclature proposed by Rivailler et al^16^) were retrieved from the GenBank using the BLAST program in NCBI. All study sequences, WHO reference sequences and sublineage reference sequences were aligned using the ClustalW alignment program within the Molecular Evolutionary Genetics Analysis (MEGA7) software.^17^ The phylogenetic trees were inferred using the maximum likelihood method based on the Tamura‐Nei model. The robustness of the nodes was tested with 1000 bootstrap replications and bootstrap support values greater than 75 are shown at the nodes. Rubella sequence data from this study were deposited in the GenBank with the accession numbers MK399390‐MK399397.

#### 2.2.4 Epidemiological analyses

All the data obtained, that is, both demographic and laboratory results were entered in an electronic database. The districts were grouped into the administrative regions as provided by the Uganda Bureau of Statistics. For this publication, data were analyzed using EpiInfo version 3.3.2 and tables showing seroprevalence by sex, age, region, and month generated.

## 3 RESULTS

### 3.1 Patient demographics

Out of the 5334 rubella IgM‐positive cases, 2710 (50.8%) were females compared with the 2624 (49.2%) males. Among the female rubella cases, 2609 (96.3%) cases were below the age of 15 years with the remaining 101 (3.7%) above the age of 15 years. The Central region of the country had the highest number of confirmed cases (2036 out of 5334; 38.2%), followed by the Western (1542 out of 5334; 28.9%), Eastern (1036 out of 5334; 19.4%) with the Northern region (720 out of 5334; 13.5%) having the least number of confirmed cases. Table 2 summarizes the patient demographics.

**Table 2:** Patient demographics

Patient demographics	Year													
	2005	2006	2007	2008	2009	2010	2011	2012	2013	2014	2015	2016	2017	2018*
Male (11 763)	543	1180	1014	733	627	670	945	958	600	803	1370	716	590	1014
Female (11 433)	485	1125	938	817	558	624	873	946	588	837	1309	706	606	1021
Age group of investigated cases														
<5 (13 734)	566	1504	1030	855	711	805	1049	1097	734	1059	1517	917	706	1184
≥5 to <10 (6386)	299	491	579	484	332	341	563	547	301	430	782	361	352	524
≥10 to <15 (2093)	100	161	220	169	111	101	162	176	87	108	279	119	112	188
≥15 (983)	63	149	123	42	31	47	44	84	66	43	101	25	26	139
Age group of all rubella IgM‐positive cases (females only)														
<5 (2285 (1133))	115 (53)	134 (67)	205 (98)	185 (93)	62 (30)	53 (20)	244 (123)	193 (99)	80 (44)	143 (77)	511 (234)	126 (67)	117 (64)	117 (64)
≥5 to <10 (2165 (1090))	140 (59)	131 (75)	250 (119)	211 (123)	87 (34)	55 (24)	252 (122)	199 (97)	60 (39)	113 (59)	373 (189)	108 (43)	103 (55)	83 (52)
≥10 to <15 (705 (386))	37 (21)	45 (25)	119 (61)	73 (37)	26 (13)	21 (10)	65 (34)	64 (37)	16 (11)	31 (22)	120 (69)	36 (16)	32 (17)	20 (13)
≥15 (179 (101))	12 (06)	14 (09)	38 (17)	15 (09)	06 (04)	02 (01)	13 (07)	17 (12)	05 (03)	08 (06)	31 (21)	07 (02)	05 (03)	05 (01)
Regional distribution of investigated cases														
Central (10 211)	583	1327	943	697	543	707	895	796	488	750	1000	482	394	606
Eastern (5145)	191	410	418	305	299	336	513	380	210	298	514	419	317	535
Northern (2408)	63	103	95	139	146	88	123	208	117	155	324	212	203	432
Western (5432)	191	465	496	409	197	163	287	520	373	437	841	309	282	462
Regional distribution of rubella IgM‐positive cases														
Central (2035)	166	188	266	213	79	58	267	160	36	134	347	43	33	45
Eastern (1037)	35	45	134	78	32	46	158	67	10	35	174	92	65	66
Northern (720)	32	25	18	49	51	10	43	68	24	30	167	70	79	54
Western (1542)	71	66	194	145	19	17	106	178	91	96	347	72	80	60

Abbreviation: Ig, immunoglobulin.

* Data upto July 2018.

### 3.2 Spatio‐temporal distribution of rubella IgM‐positive cases

**Figure 1:**
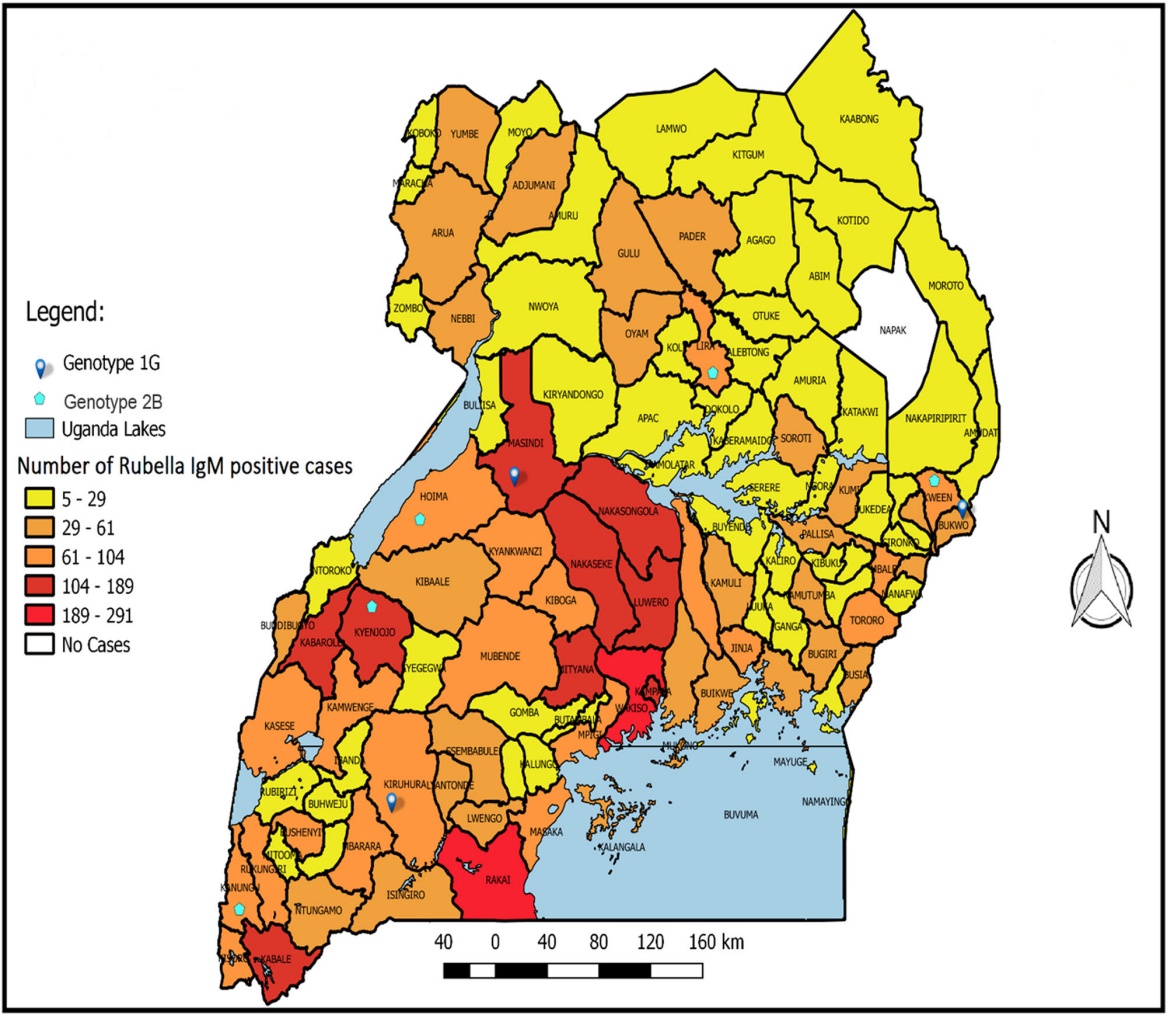
Distribution of rubella IgM‐positive cases in Uganda by district, January 2005 to July 2018, and genotypes from typed sera, January 2012 to August 2017. Ig, immunoglobulin

A total of 23 196 suspected measles cases were investigated from January 2005 to July 2018 with 5334 (23%) being rubella IgM‐positive and 2910 (12.5%) measles IgM‐positive. The rubella IgM‐positive cases were distributed throughout the country (Figure 1). Only one district (Napak district) out of the 116 did not have a confirmed rubella IgM‐positive case.

The seven districts (Napak, Moroto, Kotido, Abim, Nakapiripirit, Kaabong, and Amudat) that make up the Moroto subregion in Eastern Uganda had the lowest incidence per 100 000 population. Nakasongola district in the Mubende subregion in Central Uganda had the highest incidence per 100 000 population over this period.

**Figure 2:**
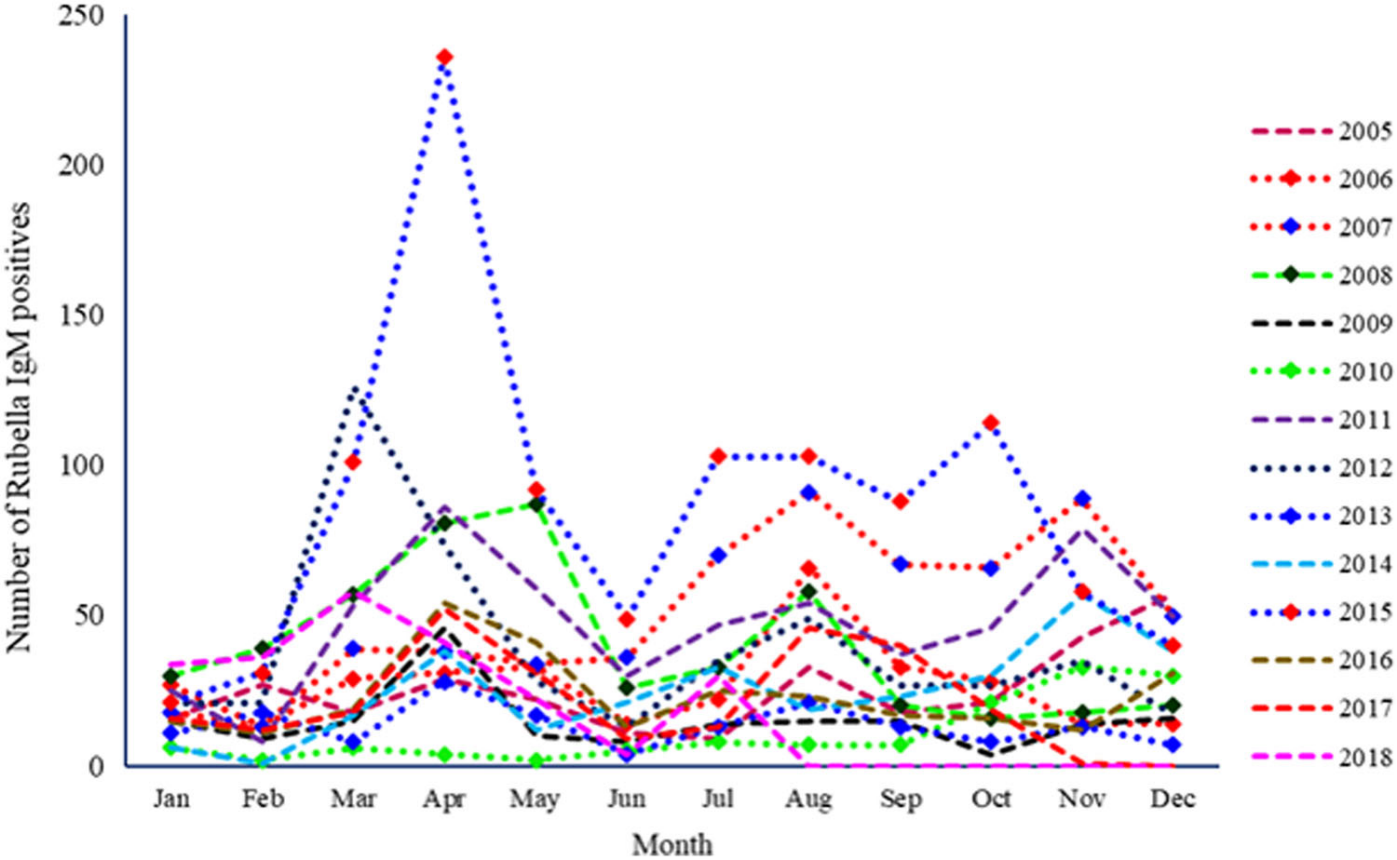
Distribution of rubella IgM‐positive cases in Uganda by month and year. Ig, immunoglobulin

Over the years, the highest number of rubella IgM‐positive cases by month of receipt in the laboratory was observed in April, August, and November (Figure 2). The year 2010 had the least number of rubella IgM‐positives (181) whereas 2015 had approximately 10 times (1036) the number seen in 2010.

### 3.3 Rubella virus sequence distribution and phylogenetic analysis

Of the 100 sera tested at CDC, 18 were positive by real‐time PCR. For eight of these, the 739‐nt window of the E1 glycoprotein was successfully amplified and sequenced. Two genotypes were detected, 1G (three sequences) and 2B (five sequences). The majority of these (5 out of 8; 62.5%) were obtained from cases whose serum had been collected within a day of rash onset. Genotype 1G was detected in 2012 and 2016 and genotype 2B in 2014, 2015, and 2017 (Table 3). The Western and Eastern regions had both genotypes 1G and 2B, the Northern region had genotype 2B and the Central region had none detected.

**Table 3:** Rubella viruses genotyped for this paper

GenBank accession number	WHO name	Year of onset	District of onset	Region	Genotype	Lineage
MK399397	RVs/Masindi.UGA/12.12	2012	Masindi	Western	1G	1G‐L2b
MK399396	RVs/Kiruhura.UGA/12.12	2012	Kiruhura	Western	1G	1G‐L2b
MK399395	RVs/Kanungu.UGA/47.14	2014	Kanungu	Western	2B	2B‐L2c
MK399394	RVs/Kyenjojo.UGA/02.15	2015	Kyenjojo	Western	2B	2B‐L2c
MK399393	RVs/Lira.UGA/43.15	2015	Lira	Northern	2B	2B‐L2c
MK399392	RVs/Bukwo.UGA/18.17	2017	Bukwo	Eastern	1G	1G‐L2b
MK399391	RVs/Kween.UGA/03.17	2017	Kween	Eastern	2B	2B‐L2c
MK399390	RVs/Hoima.UGA/18.17	2017	Hoima	Western	2B	2B‐L2c

Abbreviation: WHO, World Health Organization.

**Figure 3:**
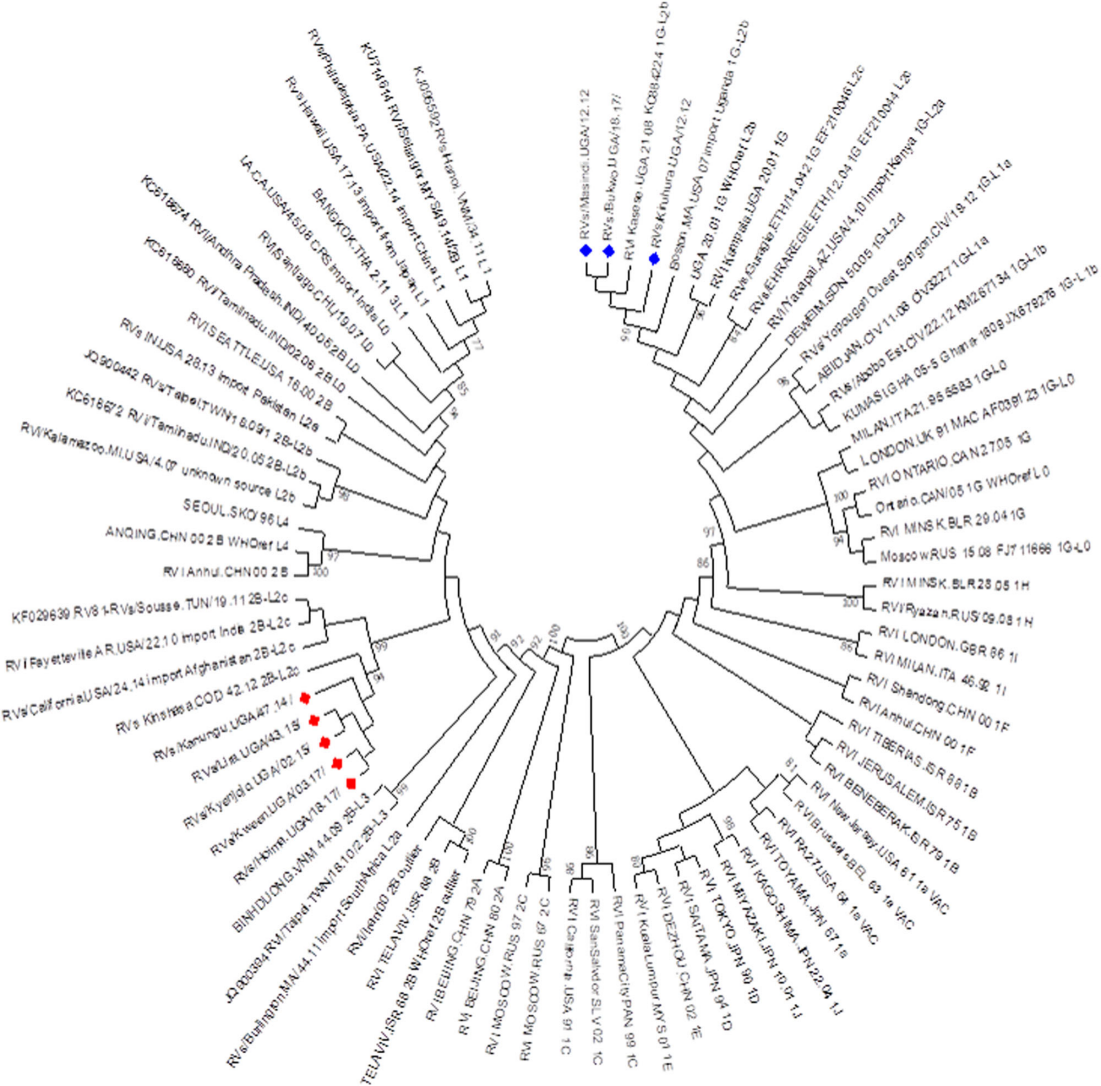
Phylogenetic tree showing the study sequences along with WHO rubella reference sequences and rubella sublineage reference sequences. Tree was inferred using the maximum likelihood method based on the Tamura‐Nei model. The robustness of the nodes was tested with 1000 bootstrap replications and bootstrap support values greater than 75 are shown at the nodes (study sequences denoted with a red [genotype 2B] or blue [genotype 1G] diamond)

Phylogenetic analysis of the study sequences along with WHO rubella reference sequences and rubella sublineage reference sequences proposed by Rivailler et al^16^ showed the genotype 1G sequences belonged to sublineage 1G‐L2b and the genotype 2B sequences belonged to sublineage 2B‐L2c (Figure 3).

## 4 DISCUSSION

The present study documents that rubella in Uganda is a childhood disease affecting mainly those below 15 years of age. This is in agreement with previously published findings.^4^, ^18^, ^19^, ^20^, ^21^ However, because the immunization program mainly targets those under 5 years of age, and current surveillance is focused on measles, under‐reporting may be high among those older than 5 years. Also, HIV‐infected infants, because of their defective cell‐mediated immunity, may not develop the characteristic measles like rash^22^, ^23^ which is the basis for the clinical diagnosis of measles (and inadvertently the diagnosis of rubella) according to the WHO standard case definition.^13^ Furthermore, 20% to 50% of all rubella infections are subclinical.^3^

The majority of the females who were rubella IgM‐positive were under the reproductive age of 15 years. This is similar to what was reported in Ethiopia, Cameroon, Zimbabwe, and Central African Republic.^18^, ^19^, ^20^, ^21^, ^24^ This very likely leads to immunity during childbearing years. According to the Uganda Demographic and Health Survey key indicators report of March 2017, 25% of adolescents aged 15 to 19 had begun childbearing at the time of the survey, 19% of women in the same age group had already given birth while 5% were pregnant with their first child.^25^ In this study, 3.7% (101 out of 2710) of the females that were rubella IgM‐positive were above 15 years of age and as such putting them at the risk of having CRS babies. A systematic search of online databases by Babigumira et al^26^ showed that the estimated cost of treatment of CRS in 2012 was between $4200 and $57 000 per case annually in middle‐income countries and up to $140 000 over a lifetime in high‐income countries with no data reported for low‐income countries. This cost is likely unmanageable for low‐income countries, and no doubt leads to limited treatment and services for affected individuals. Simons et al^27^ puts the percentage of optimally treated CRS cases at 0% and the infants who do survive live for fewer years with severe disability.

The highest number of rubella IgM‐positive cases was seen in the months of April, August, and November (Figure 2). Uganda has a tropical climate with a bimodal rainfall pattern characterized by rains in the months of March to May and October to December with the remaining months being generally dry with limited rainfall.^28^ April and November, noted to have the highest number of rubella IgM‐positives, are months characterized by rains. This is contrary to what was been reported in Zimbabwe where the highest number was seen in the late spring months (October to November) which are generally dry.^20^ Although the rainfall patterns may differ between the two countries, the months with the highest prevalence, that is, March/April and October/November are similar in many years. Also, it is interesting to note is that the rubella incidence appears to be very low for both countries in 2009 and most of 2010 (until October). This pattern suggests a multiyear cycle for rubella in the two countries. Combined data from the current and a previous report of rubella in Uganda support a 3‐ to 4‐year rubella cycle in Uganda.^8^ Goodson et al^4^ argue that a multiyear cycle is not present in the Eastern subregion of Africa but these data do not support this argument.

Rubella RNA detected in archival sera was used to obtain rubella genotypes from 2012 to 2017. The sera had been stored at −20°C for up to 5 years before testing with an unknown number of freeze/thaw cycles. Nevertheless, 18% of sera contained detectable rubella RNA and 8% yielded genotypes. This success rate is consistent with previous reports of genotyping rubella virus from archival serum (Zheng et al, 18% RT‐PCR‐positive and 7.8% genotyped; Lazar et al,^29^ 21.5% RT‐PCR positive and 7.5% genotyped) providing additional evidence that this is an effective method for retrospective genotype baseline determination for rubella virus.^10^ Zheng et al^10^ were able to obtain genotypes 1C, 1E, and 1G using these methods implying that this method is able to identify other rubella genotypes. Genetic characterization of the eight samples revealed two genotypes, 1G and 2B. Namuwulya et al^9^ identified genotypes 1G and 1E to be circulating in the country with genotype 1G being predominant. However, of the eight samples genotyped, only three were of genotype 1G and five were of genotype 2B. The genotype 1G sequences belonged to sublineage 1G‐L2b along with previous sequences from Uganda. Rivallier et al^16^ previously reported a geographical clustering of 1G‐L2 sublineages with 1G‐L2a found in Kenya, 1G‐L2b in Uganda, 1G‐L2c in Ethiopia, and 1G‐L2d in Sudan. However, unpublished data detected sublineage 1G‐L2c in a 1‐month‐old CRS baby in 2015, implying the circulation of different genotype 1G sublineages in the country. All genotype 2B sequences belonged to sublineage 2B‐L2c. This is the first report of genotype 2B in Uganda; this genotype 2B virus lineage was initially identified in 2014 with subsequent identification in 2015 and 2017, suggesting that transmission of this lineage has become established within Uganda. The appearance of genotype 2B‐L2c is consistent with patterns seen in other African countries in recent years. In the Democratic Republic of Congo, viruses of genotype 2B‐L2c were first detected in 2012^11^ and in Côte d'Ivoire, only genotype 1G was found before 2016 when a virus of genotype 2B‐L2c was identified.^30^ Although the baseline data from most countries in sub‐Saharan Africa are limited, the observation of the same pattern in several countries, suggests that viruses of genotype 2B‐L2c are a recent introduction into Central and Eastern Africa and that multiple introductions or intercountry spread is occurring. Continuous virological surveillance, with the goal of identifying virus genotypes from each outbreak of rubella should be maintained in Uganda. Informed knowledge of rubella viruses circulating in Uganda will enhance efforts to monitor the impact of the vaccination strategy as Uganda moves toward control and elimination of rubella and CRS.^31^

## 5 LIMITATIONS

This study used samples collected during the routine measles surveillance. In Uganda, routine measles surveillance targets children under the age of 15 that present with rash and fever. Therefore, some rubella cases especially the subclinical ones and those above 15 years of age may have been missed.

Secondly, genotyping from sera requires acute phase specimens. This is because the highest viral titers in blood typically occur before rash onset,^14^ which makes timing of sample collection critical. In this study, all the specimens that were successfully genotyped had been collected within 2 days of rash onset. Nonetheless, the use of sera is intended to supplement already existing molecular surveillance methods.

In addition, the study used archived samples that had been stored at −20°C (as opposed to the recommended −70°C for preservation of RNA) and could have undergone a series of freeze/thaw cycles. The combination of long storage at −20°C and unknown freeze/thaw cycles may have had a deleterious effect.
